# Extended Wrist Rotation Simplified

**DOI:** 10.7759/cureus.54319

**Published:** 2024-02-16

**Authors:** David G Changaris

**Affiliations:** 1 Neurological Surgery, Independent Researcher, Louisville, USA

**Keywords:** cogwheel rigidity, muscular rigidity, dystonia upper extremity, adult-onset dystonia, tremor dystonia, parkinson' s disease, parkinson-plus syndromes

## Abstract

Extended wrist rotation provides a simple clinical measure of rigidity in movement disorders. The supinator-pronator muscles of the forearm form an agonist-antagonist pair that can be isolated for diagnosis and monitoring. Patients rarely can isolate these muscles without extraordinary training and body awareness. Clinicians may find documenting the impact of the shoulder girdle, wrist, and hand movements overburdensome. A preliminary study shows that restricting the olecranon and keeping the wrist in line with the hand can provide a simple, reproducible measure of rigidity. We study a two-handed “handshake” examination and the use of a pulley-based goniometer. This preliminary analysis indicates that both offer the same observer and between-observer reliability. Two-way analysis of variance showed no statistical differences or outliers.

## Introduction

Agonist-antagonist paired muscles contribute to the rigidity of dystonia and movement disorders [1]. Ordinarily, one of the paired muscles contracts while the other relaxes. When the antagonist muscle fails to relax rigidity develops. Not only does this cause movement disorders in a specific body part, but electrical evidence of this extends to other "non-dystonic body parts at a subclinical level" [2]. In his study, de Vries called this condition a "pre-dystonic state" [2]. 

In clinical rigidity the relaxation of the antagonist fails, reducing the loss of the range of motion. The supinator-pronation of the extended wrist has clinical value in defining rigidity [3]. Unfortunately, this “low tech” observation rarely finds utility beyond the movement specialist neurologist. Clinical observation of rapid alternating pronation-supination contributes to the Unified Parkinson’s Disease Rating Scale (UPDRS) [4].

Parkinson’s disease has seen a doubling in young adults over the five years (2012-2017) [5]. The increasing prevalence of Parkinson’s disease [6] multiplies the added risk of the surgical morbidity the Parkinsonian patient brings to surgical procedures [7,8]. Having an accessible, reliable clinical diagnostic examination would help non-movement specialists identify these patients to improve post-operative outcomes. Rarely, surgery may be seen as the proximate cause or worsening of an underlying dystonia [9,10].

The National Institutes of Health in April 2001 held a workshop to define the terms "spasticity," "dystonia," and "rigidity." The meeting concluded and published, "Rigidity" is defined as hypertonia in which all of the following are true: 1) the resistance to externally imposed joint movement is present at very low speeds of movement, does not depend on imposed speed, and does not exhibit a speed or angle threshold; 2) simultaneous co-contraction of agonists and antagonists may occur, and this is reflected in an immediate resistance to a reversal of the direction of movement about a joint; 3) the limb does not tend to return toward a particular fixed posture or extreme joint angle; and 4) voluntary activity in distant muscle groups does not lead to involuntary movements about the rigid joints, although rigidity may worsen" [11].

The extended wrist rotation is one of three motor tasks from the Unified Parkinson's Disease Rating Scale (UPDRS part III, pronate-supination movement of the hands, task 25). During the pronate-supination task, subjects are asked to extend the arm out in front of them with the palms down and then to turn the palm up and down alternately 15 times, as fast and as fully as possible [11].

Dystonia may show involuntary muscle contractions in the wrist and hand, leading to abnormal movements or postures. Common symptoms include twisting of the wrist, flexion or extension of the fingers, and difficulty controlling wrist movements [3]. During the disability determination, the loss of wrist rotation while the elbow is bent defines a graded "whole person impairment" as specifically outlined by Cocchiarella and Anderson [12]. This impairment measure uses the “bent-elbow” method. The elbow bent 90 degrees and held close to the chest, allows the forearm, parallel to the ground, to rotate externally (supinate) and internally (pronate). Fractures and surgical procedures can physically limit wrist rotation. The combined normal range for pronation and supination from the vertical adds to 160 degrees and above. With the elbow bent, additional muscles participate in wrist rotation. The biceps brachii inserts on the radial tuberosity and contributes to supination with the elbow bent. The brachialis, which extends from the humerus to the wrist, also participates.

Motor symptoms of Parkinson’s disease include bradykinesia, resting tremors, and rigidity [11]. In Parkinson's disease, loss of substantia nigra cells probably starts long before symptoms develop; in fact, an estimated 80% depletion of striatal dopamine occurs before the onset of motor symptoms. Parkinson’s disease remains a clinically defined condition. The impact has created a breathtaking range of complex diagnostic tools available to affirm this still incurable condition. In limb dystonia the pattern EMG identifies confirming patterns consistent with dystonic rigidity [13].

Today, movement studies incorporate complex inertial and geomagnetic sensors to capture total body movement and individual body parts. These systems are costly in terms of physical equipment, space requirements, and user training [14]. A simple clinical measure to document rigidity may serve as a sentinel for further testing or early intervention to prevent progression. Having an accessible screening tool, if reliable within and between observers, might allow others besides movement-specialist neurologists to track the efficacy of clinical interventions.

## Technical report

**Figure 1:**
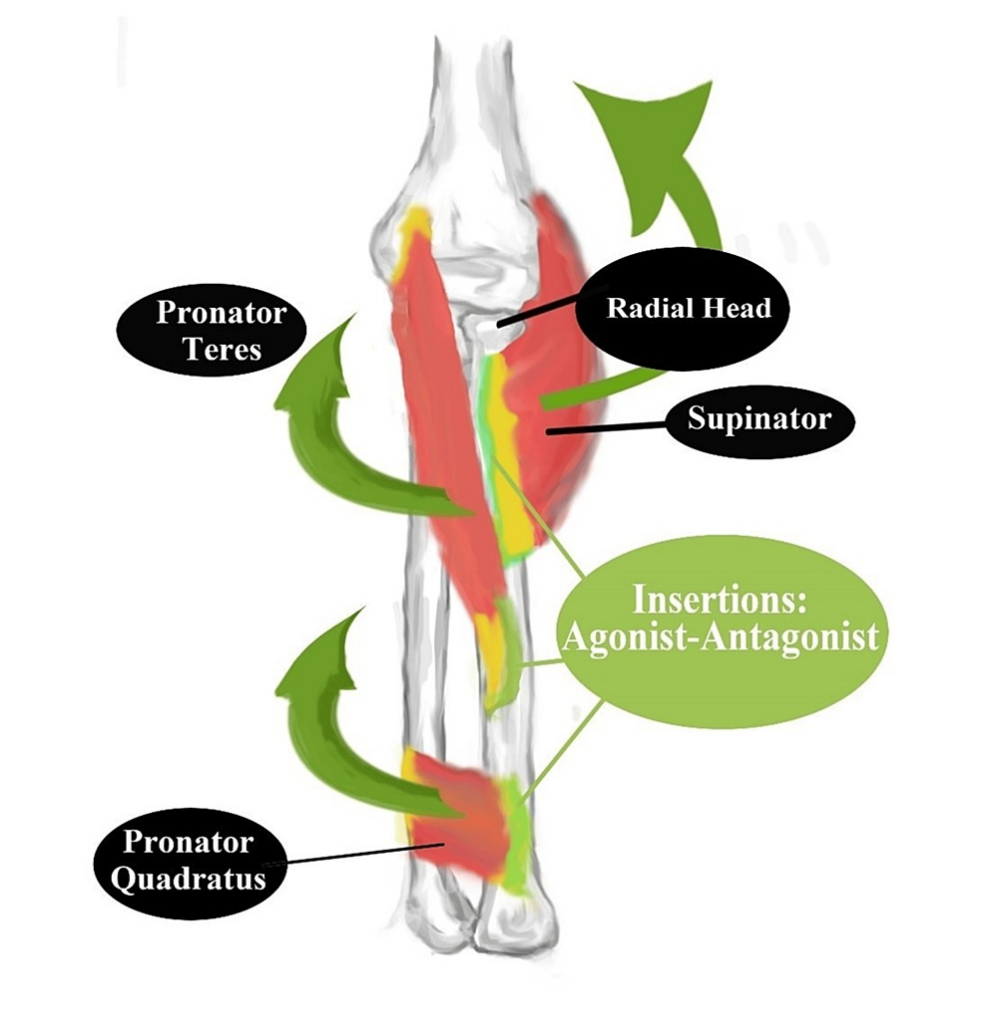
Dominant agonist-antagonist muscles within the forearm The forearm supinator muscle inserts on the proximal 1/3 of the radial bone. The pronator teres inserts on the opposing surface of the mid-radial bone. These form an agonist-antagonist pair for early rigidity assessment. Immobilizing the olecranon and wrist during extended wrist rotation isolates these muscles to allow a reproducible measure. Tenderness along the radial insertions (light green) with normal exam elsewhere points to rigidity during extended wrist rotation. This digital drawing was created by the author.

Figure 1 shows the forearm supinator and pronators dominant in the extended wrist rotation. In the normal human, these rotate the wrist sometimes exceeding 160-180 degrees. When this excursion fails specific medical and traumatic conditions shown in Table 1 may be the cause. The absence of tenderness along the epicondyles and related nerves passing the upper to lower arm provides the setting where rigidity lends to considering dystonia and Parkinson's disease. The patient with apparent loss of range of motion of the extended wrist needs a full review of the conditions that cause pain to the forearm. These include the diagnoses shown in Table 1.

**Table 1:** Diagnoses associated with tenderness of the forearm within medial and lateral to the olecranon and the wrist. The table conditions may be present within the common sites of reported pain. Rheumatoid arthritis, osteochondritis dessicans, sprains, bursitis, and tumors will also affect the forearm.

Medial to Olecranon	Lateral to Olecranon	Wrist
Cubital tunnel syndrome	Posterolateral ulna instability	Carpal tunnel syndrome
Medial epicondylitis	Lateral epicondylitis	Ganglion cysts
Golfer’s elbow	Tennis elbow	Intersection syndrome
	Lateral ulnar collateral ligament Injury	Kienböck's disease

Analysis for isolating wrist rotation

Rarely in clinical settings can the patient voluntarily isolate specific muscles. Because the clinical observer cannot easily separate the simultaneous contributions of wrist flexion and shoulder rotation, limiting these within a protocol or device creates a working tool.

Looking more proximally, the humerus (upper arm) within the glenohumeral joint (shoulder) is rotated externally by the infraspinatus and teres minor. The upper arm's internal rotation depends upon the subscapularis, pectoralis major, latissimus dorsi, teres major, and the anterior aspect of the deltoid. Both can be restricted while preserving wrist rotation by flexing the elbow and holding the elbow close to the lateral chest. Alternatively, securing the olecranon and wrist allows the extended wrist. This study measures the effect of limiting the elbow and wrist by a human and by a device. With the extended arm, the wrist rotation measure minimizes the biceps and extensor longus impact.

At the extremes of wrist rotation, flexing the wrist may cause the wrist to pronate 10-20 degrees. Disease and injury of the wrist impact this. Extending the wrist and forearm while immobilizing the olecranon allows for visually assessing the effect of this wrist flexion. Using a 2-inch diameter rod for the hand to grip reduces the tendency to flex the wrist during rotation. Careful visual assessment during the wrist rotation measures can detect inadvertent attempts to flex the wrist or involve intrinsic muscles of the hand. Similarly, placing the arm and hand in the fully extended position allows for minimizing the impact of shoulder rotation and abduction. If one holds the hand in the plane of the wrist, abducting or adducting the shoulder can rotate the wrist 40 degrees or more. Not controlling this creates unpredictable results even with small shoulder movement.

Two-handed “Handshake” examination

**Figure 2:**
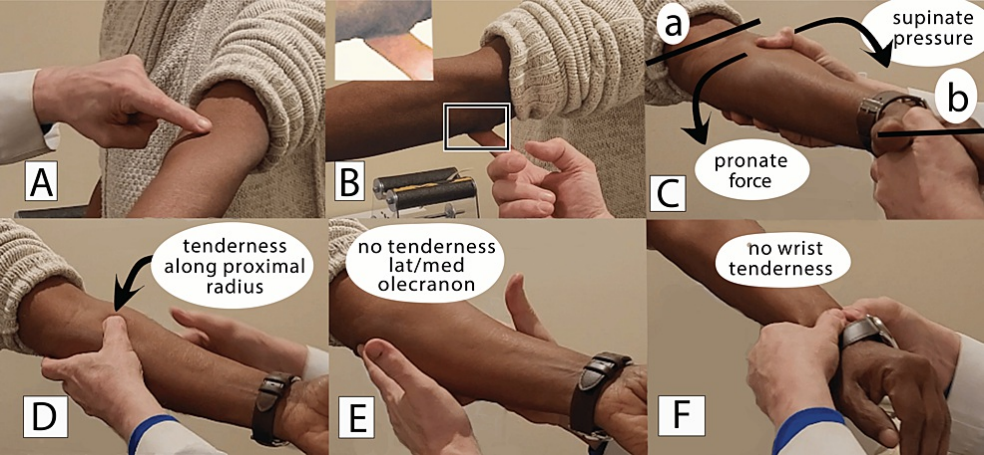
Instructional sequence showing the "Handshake" measurement of extended wrist rotation In Figure 2A, the patient is instructed to imagine an “eye” looking straight up at the ceiling and keep this position. In Figure 2B, the observer places the index finger on the olecranon to grip the supinator. Gentle 1-2 lbs. of pressure applied to the supinator reminds the patient to keep the elbow “fossa” pointing to the ceiling. In Figure 2C, the other hand of the clinician engages in a “handshake” to follow the hand-wrist complex during supination-pronation. This pulls the hand and wrist into alignment. The two hands establish a line paralleling the distal radius and ulna within the wrist (Figure 2C Line a and Line b.) When there is rigidity within the forearm muscles, the patient will begin to rotate the plane of the “eye” before reaching the normal range, as can be seen (Figure 2C). Line Figure 2Ca correlates to the elbow fossa and has begun clear rotation. The distal Line Figure 2Cb has failed to become parallel to the ground. Again, this angle between lines 2Ca and 2Cb (just before line 2Ca begins to move) is the range of rotation of the extended wrist. The clinician can discern a pulling pressure against the distal thumb when the plane of the “eye” rotates. At the beginning of this rotation, one can easily assess the angle between the line of the elbow (Figure 2Ca) of the distal radius and ulna (Figure 2Cb). This determines the wrist rotation motion discussed herein. Concomitantly, significant tenderness can often be evoked along the proximal medial radius shown (Figure D). Often, the patient is not aware of this proximal-radial tenderness. The palpation of the medial and lateral elbow regions has no tenderness, as shown (Figure 2E). The absence of wrist tenderness is shown (Figure 2F). Photographs were taken by Leslie Patterson and edited by the author.

Figure 2 shows the clinician using one hand to engage the patient’s hand as if greeting the patient (Figure 2C). The other hand places the fingers on the olecranon to gently immobilize the extended elbow. Placing the thumb upon the supinator reminds the patient to limit the excursion of the elbow. The patient is instructed to imagine an “eye” in the crease of the elbow “looking straight up at the ceiling.” The patient is asked to keep the “eye looking up while rotating the wrist.” During active wrist rotation, the examiner maintains a firm grip pressure of 1-2 lbs. The clinician documents the presence of tenderness pointing to common painful sources listed in Table 1, e.g., within the medial or lateral olecranon region or wrist. Switching hands and positions allows measurement of the other arm.

Goniometric measurement of extended wrist range of motion

**Figure 3:**
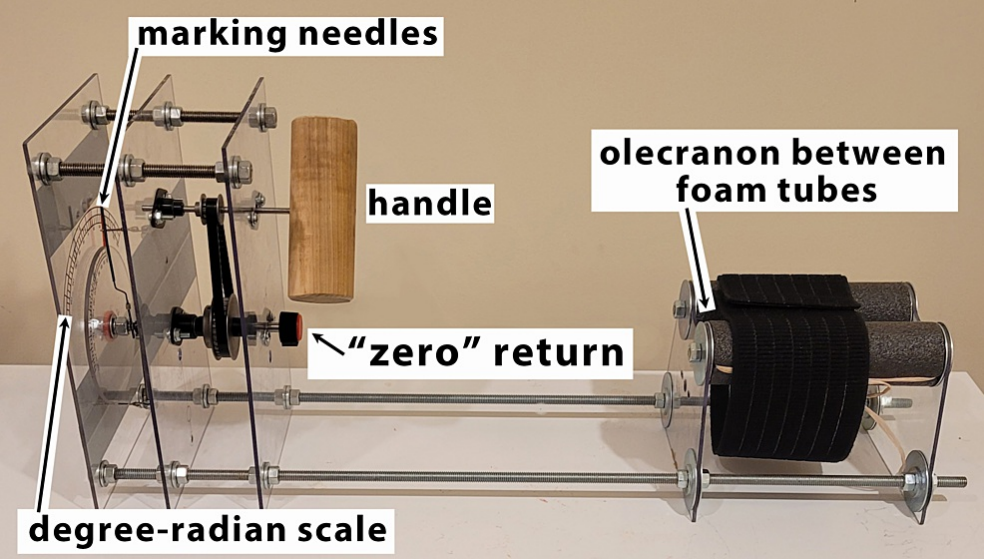
Goniometer for extended wrist rotation The image shows a “goniometer” for extended wrist rotation. The olecranon is palpated within the space between the gray foam tubes. A cloth gently holds the forearm in position.  The patient holds and turns the handle to move the needles medially and laterally for establishing the range of motion. The photograph was taken and edited by the author.

While the allure of the expensive and complex remains, the simple mechanical goniometer described herein provides a measure of the extended wrist rotation. Minimal costs of parts and labor make this device accessible. Figure 3 shows the tool used for this measurement.

**Figure 4:**
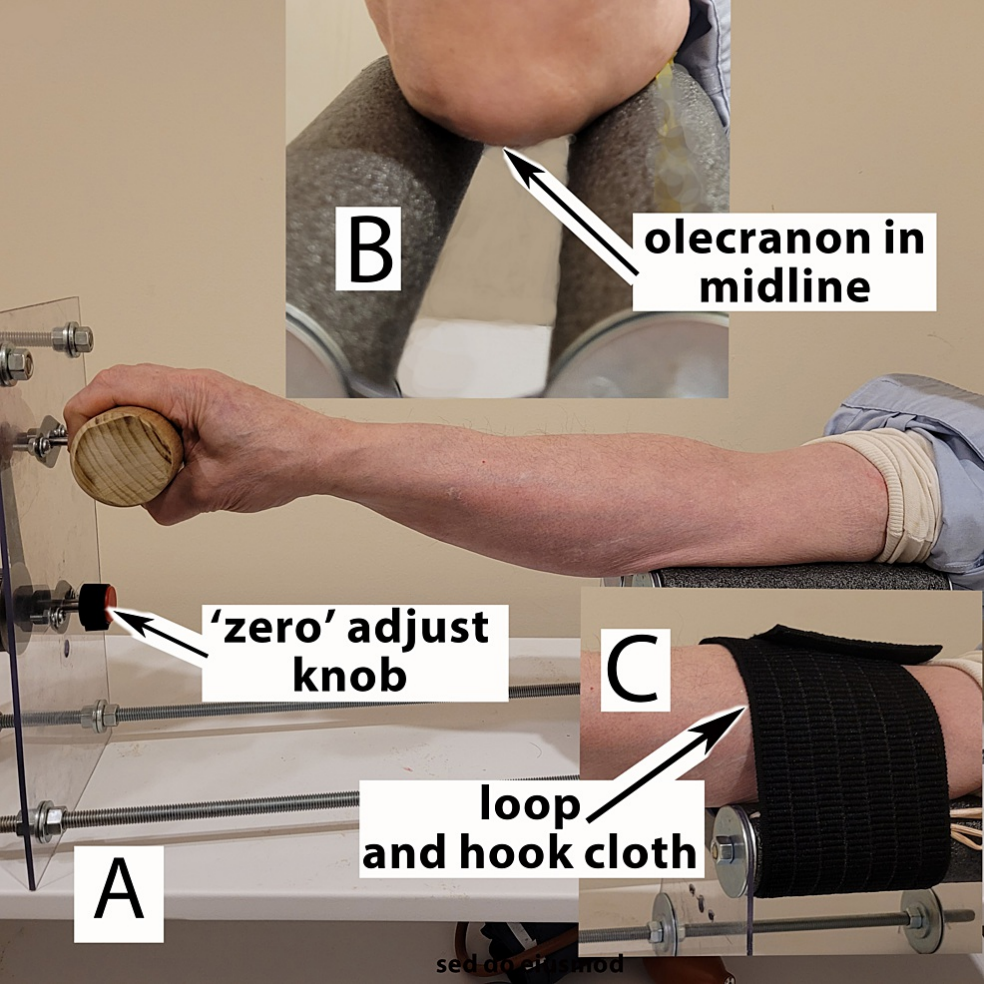
Goniometer shown with arm in place The olecranon rests in the middle of the two gray foam cushions (Figure 4B). The hand grips the 2" dowel to stretch the wrist (Figure 4A). This stretching limits the impact of unintentional wrist flexion. Figure 4C shows the "loop and hook cloth" that gently reminds the patient to hold position while rotating the wrist. Figure 4A shows the "zero adjust knob" that resets the goniometer. The photograph was taken by Leslie Patterson and edited by the author.

The patient is seated comfortably with the elbow on the two pads within the goniometer (Figure 4). The clinician palpates the olecranon between the two foam tubes. The proximal arm is gently immobilized with a “hook-and-loop” cloth belt. The forearm extends to grasp the 2-inch diameter dowel. The dowel connects to a metal rod that rotates needles hidden from the patient. The circular rotation “internally” and “externally” may be separately or jointly measured on the “degree-radian scale.” The device has rotational knobs to return the moving arms to “zero.” The patient cannot see the results of rotating measuring needles.

Statistical design

Four clinical observers made five separate measures by “Handshake” and by “Goniometer” using the extended wrist rotation protocols on the same patient. This format lent to a basic repeated measures analysis of variance testing using a right-tailed F distribution. The combined normal range of motion reported in the AMA Guides to the Evaluation of Medical Impairment (Fifth Edition) for bent elbow lists as 160 Degrees [12]. This study incorporated this as the “normal” range. The onset of concern or smallest loss of rotation begins at 150 degrees or a 10-degree loss of combined supination-pronation. The subject patient had developed clinical evidence of a disordered extended wrist rotation. A 10-degree variance was proposed as significant with a 160-degree normal range of supination and pronation [12].

Results

The measurement subject was a 68-year-old female. She had noted the new onset of loss of coordination in her right hand two years after a motor vehicular accident. In this accident, she sustained a mild traumatic brain injury evidenced by vertigo of central origin on videonystagmography and balance disorder on computerized posturography [15,16]. She had been treated with physical and balance therapies. She claimed subtotal relief from her symptoms of dizziness. She continued to complain of worsening general anxiety since the accident. Palpation of the right supinator muscle caused significant reported pain along the proximal radius. She had no tenderness along the medial and lateral olecranon/epicondyles for 3-4 inches distally from them. Her wrist was non-tender. The patient consented to allow four staff members to engage in repeated measures as part of our internal quality control and training. She reported no untoward discomfort afterward. Her benefit was a measure validated by four licensed medical personnel (one Registered Nurse, one Licensed Practical Nurse, one Physical Therapist, and one Physician).

**Table 2:** Average measures from four clinicians using both "Handshake" and "Goniometer" methods on the same patient The table shows averages for the four clinician observers. The "Handshake" method showed an average of 145 degrees; the "Goniometer" showed an average of 143 degrees. The repeated measure analysis of variance identifies that the four observers showed no intra-observer or between-observer significant differences (the null hypothesis was accepted after the two-way repeated-measures analysis of variance). The p-value equals 0.098 (p(x ≤ 2.9) = 0.9) and indicates no significant differences present. This affirms the null hypothesis.

Observer	Handshake	Goniometer	Average
1	146	143	144
2	145	140	143
3	146	143	145
4	144	146	144
Average	145	143	144 Grand

The analysis of variances shows no statistical differences between or within groups. The null hypothesis fails to be rejected as the p-value is greater than α=0.05. The differences between the “handshake” and the “goniometer” measures are too small to be statistically significant. The p-value equals 0.09777, (p (x ≤ 2.9091) = 0.9022). The calculated F equals 2.9, which is in the 95% region of acceptance: [-∞, 4.1]. The size effect and partial size effect have limited value, considering observations were made with one patient and four observers [17]. Both were “medium-sized.” There are no outliers (Tukey Fence, K=1.5), and the Power of the test is 1, making it unlikely that any of the groups are different (rejecting the H0). With two groups and one difference, the variances of the groups are assumed to be normally distributed (sphericity).

## Discussion

This simple, rapid measure of extended wrist rotation provides inter and within-observer reliability within a small testing sample on a single patient. Both the “Handshake” and “Goniometer” methods will need further testing to validate for specific functions. While completing this measure, the clinician can assess the range of motion and identify joint stiffness, contractures, and/or limb disease.

Because understanding has evolved that even a single brain injury increases the pooled-odds ratio for Parkinson’s disease, Alzheimer’s disease, and amyotrophic lateral sclerosis [18], a large pool of people remains at risk for early onset of symptoms. Subsequent studies corroborate this [19,20]. Knowing that neurodegeneration increases in mild traumatic brain injury creates a need for a simple clinical measure of rigidity. Our subject suffered a traumatic brain injury two years before these measures. The rigidity shown in the testing evolved since her accident. Ordinarily, ‘parkinsonism’ implies the presence of one of the symptoms of Parkinson’s disease such as rigidity. Rigidity, as measured herein, offers a clinically measurable tool in the “pre-dystonic state.” 

In the absence of structural pathology, focal tenderness of joint inflammation, and/or nerve entrapments reduced extended wrist rotation points towards rigidity (dystonia/parkinsonism). Slow movements, resting tremors, and loss of postural reflexes are other motor impairments that support the diagnosis of Parkinson’s disease. When present, rigidity, as measured herein, may provide a tool to evaluate therapeutic interventions within the spectrum of dystonia/parkinsonism/Parkinson’s Disease.

## Conclusions

This study employed repeated measures of a single patient. While statistically sound, conventional guidance for human clinical study requires many patients to be tested. This paper, with its strong statistical data, offers support for more extensive testing. From the ongoing process of developing this device, the measure of the extended wrist rotation offers depth to documenting progressive impairment.

Finding subclinical midline forearm tenderness along the proximal radius has provided an early clinical marker for rigidity. Often, patients are not aware of the intensity of the tenderness. Carefully showing the patient the presence of this tenderness and an asymmetric limited range of motion often creates a "teachable moment." Progressive loss of extended wrist rotation can easily be documented in an office setting. When facing a patient with arm pain not fitting a classic radicular pattern, loss of range of motion within the shoulder and wrist coupled with midline forearm tenderness, the clinician may find value in the diagnosis of rigidity (preclinical dystonia).
